# Winter Climate of St. Mary’s

**Published:** 1845-06

**Authors:** Alfred Cook

**Affiliations:** Delphi, Onondagua Co., New York


*Winter Climate of St. Mary's.* By ALFRED COOK, M. D., of Delphi, Onondagua Co., New York. Communicated by Professor DUNGLISON.

[The Author of the following notice, in consequence of the state of his health, was recommended by Dr. Dunglison to pass the winter months in the southern part of the United States. Although he returned earlier than was advisable, his condition has unquestionably been benefited by his residence there. For the advantage of others, he has furnished his impressions in regard to the place, which he chose for his winter retreat. St. Mary's is situate on the Georgia side of the river of the same name. Lat. 30°, 42′: Long. 4°, 48′ W. from Washington.]

I arrived here on the 15th of Nov., 1844, and being pleased with the situation of the place, concluded to make it my residence for a few days at least. I selected for a boarding house, an extensive well built establishment, with a large piazza in front and rear, and an extensive orange grove in the back ground, with elegant walks through it, protected by hedges, which have made it a very agreeable place through the winter for invalids to exercise in in unpleasant weather.

St. Mary's is pleasantly situated on St. Mary's river, about twelve miles from the ocean, and is well laid out. The streets are broad and intersect at right angles. The walks are generally pleasant, being covered with grass, which indicates that it is not a place of much business, and such is the fact. It contains a population of some two hundred white inhabitants, but there is a great lack of industry. The country around is a sandy plain, covered with yellow pine, oak and palmetto, with occasionally a settler. It is said to be very healthy, no case of fever having originated here for several years. Many of the inhabitants own plantations at a distance, but have selected this as a residence for their families.

The place has a supply of good water, which is said to be impregnated with iron. There are also sulphur springs in the vicinity.

Our diet has been principally fish, oysters, fowls, beef, pork, and vegetables of almost all kinds, either raised here, or brought from the north. Oysters and fish are plentiful, and many of the inhabitants subsist almost entirely on them; but, notwithstanding there is a great variety of food, the market is very irregular. Sometimes for days you cannot obtain the articles you wish, and you are compelled to eat what is set before you, which is none of the best. This, however, is universally the case south of Savannah; and although you pay from $17.00 to $25.00 per month for board, it is not good; and I think there are inducements for the establishment of good houses. The facilities for reaching St. Mary's are good, as two steam boats run between Savannah and Pulatki by the way of St. Mary's, weekly.

The climate of St. Mary's is better than I anticipated. It is midway between hot and cold;—not so warm as to be debilitating, nor so cold as to prevent exercise. In the summer, it is said to be the most agreeable residence in the South, on account of the gentle sea breeze which springs up about ten o'clock. This I enjoyed for a few days after my arrival; and then, in the evening, a breeze sets in from the land, making the nights comfortable in this warm region. But that which most delighted me in the climate, was the great amount of pleasant sunshine, which far exceeded my anticipation.

I formerly supposed that their winters here were very damp and rainy; but this has not been the case during the present winter, and I am told by physicians and other inhabitants of the place that this is never the case. There have been—as will be seen by the annexed table—but very few rainy days during the winter. Generally the storm commences in the night, and clear up in the morning; but, a few times, the storm has continued longer. All the rain, however, that fell during the winter, would not more than thoroughly wet the ground in a dry time at the north. Although there has been much north-easterly wind during the winter, it was not severe until March, which was much the most unpleasant month;—the wind being north-east much of the time, and blowing briskly.

The following table shows the state of the weather from the first of January to the tenth of March:

| Jan. | Ther. at 8 A.M. | Wind. | Pleasant fair days | Ther. at 1 P.M. | Ther. at 6 P.M. |
|---|---|---|---|---|---|
| 1 | 52 | W. | P. | 70 | 64 |
| 2 | 52 | W. | P. | 75 | 65 |
| 3 | 54 | N. E. | P. | 76 | 66 |
| 4 | 54 | N.W. | P. | 72 | 66 |
| 5 | 60 | N. E. | P. | 75 | 70 |
| 6 | 66 | W. | | 76 | 66 |
| 7 | 60 | W. | | 66 | 62 |
| 8 | 48 | W. | P. | 62 | 50 |
| 9 | 40 | W. | P. | 63 | 52 |
| 10 | 45 | N. E. | P. | 70 | 66 |
| 11 | 56 | S.W. | P. | 66 | 52 |
| 12 | 50 | W. | P. | 56 | 52 |
| 13 | 42 | W. | P. | 64 | 62 |
| 14 | 52 | W. | P. | 68 | 56 |
| 15 | 56 | N. E. | P. | 68 | 62 |
| 16 | 60 | N. E. | P. | 76 | 78 |
| 17 | 66 | W. | P. | 76 | 72 |
| 18 | 68 | S.W. | | 70 | 68 |
| 19 | 59 | N. E. | P. | 56 | 52 |
| 20 | 46 | E. | | 54 | 50 |
| 21 | 50 | W. | P. | 60 | 60 |
| 22 | 48 | N.W. | P. | 56 | 54 |
| 23 | 62 | E. | | 66 | 64 |
| 24 | 62 | W. | | 68 | 62 |
| 25 | 42 | W. | P. | 54 | 52 |
| 26 | 38 | W. | P. | 68 | 61 |
| 27 | 38 | W. | P. | 68 | 58 |
| 28 | 42 | N. E. | P. | 72 | 62 |
| 29 | 54 | W. | P. | 60 | 58 |
| 30 | 45 | N. | P. | 65 | 58 |
| 31 | 42 | N.W. | P. | 60 | 52 |

| Feb. | Ther. at 8 A.M. | Wind. | Pleasant fair days. | Ther. at 1 P.M | Ther at 6 P.M. |
|---|---|---|---|---|---|
| 1 | 44 | W. | P. | 68 | 62 |
| 2 | 52 | W. | P. | 54 | 48 |
| 3 | 50 | N. E. | | 68 | 62 |
| 4 | 58 | W. | | 56 | 48 |
| 5 | 38 | W. | P. | 50 | 44 |
| 6 | 34 | W. | P. | 54 | 46 |
| 7 | 40 | N.W. | P. | 60 | 56 |
| 8 | 40 | W. | P. | 64 | 62 |
| 9 | 48 | W. | P. | 66 | 60 |
| 10 | 46 | W. | P. | 68 | 60 |
| 11 | 46 | W. | P. | 72 | 64 |
| 12 | 58 | W. | P. | 74 | 70 |
| 13 | 59 | W. | P. | 74 | 70 |
| 14 | 58 | E. | | 75 | 70 |
| 15 | 69 | S.W. | P. | 75 | 74 |
| 16 | 50 | W. | P. | 63 | 60 |
| 17 | 46 | W. | P. | 60 | 64 |
| 18 | 50 | E. | P. | 76 | 68 |
| 19 | 52 | W. | P. | 74 | 64 |
| 20 | 58 | E. | P. | 78 | 65 |
| 21 | 64 | N. E. | P. | 81 | 70 |
| 22 | 66 | E. | P. | 80 | 74 |
| 23 | 70 | S.W. | P. | 78 | 70 |
| 24 | 64 | W. | P. | 71 | 64 |
| 25 | 50 | N. | P. | 72 | 64 |
| 26 | 50 | W. | P. | 73 | 64 |
| 27 | 52 | W. | P. | 72 | 60 |
| 28 | 46 | W. | P. | 64 | 55 |
| March. | | | | | |
| 1 | 43 | N. E. | P. | 70 | 60 |
| 2 | 64 | E. | P. | 78 | 58 |
| 3 | 69 | S.E. | | 82 | 70 |
| 4 | 69 | S. | P. | 80 | 70 |
| 5 | 66 | S.W. | P. | 76 | 69 |
| 6 | 54 | N. E. | P. | 75 | 64 |
| 7 | 62 | N. E. | P. | 76 | 70 |
| 8 | 68 | S. | P. | 85 | 71 |
| 9 | 70 | S. | | 84 | 72 |
| 10 | 71 | S.W. | P. | 86 | 68 |

It will be seen by the above table, that there were few days in which exercise could not be taken in the open air.

